# Supplementary material for: The causal relationship between the human gut microbiota and pyogenic arthritis: a Mendelian randomization study
Source: Front Cell Infect Microbiol. 2024 Nov 26;14:1452480. doi: 10.3389/fcimb.2024.1452480 (PMC11629706; doi:10.3389/fcimb.2024.1452480)
Supplement: Supplementary file 1 [file DataSheet1.zip › mendelian test/ebi-a-GCST90027682.csv_forest.pdf]

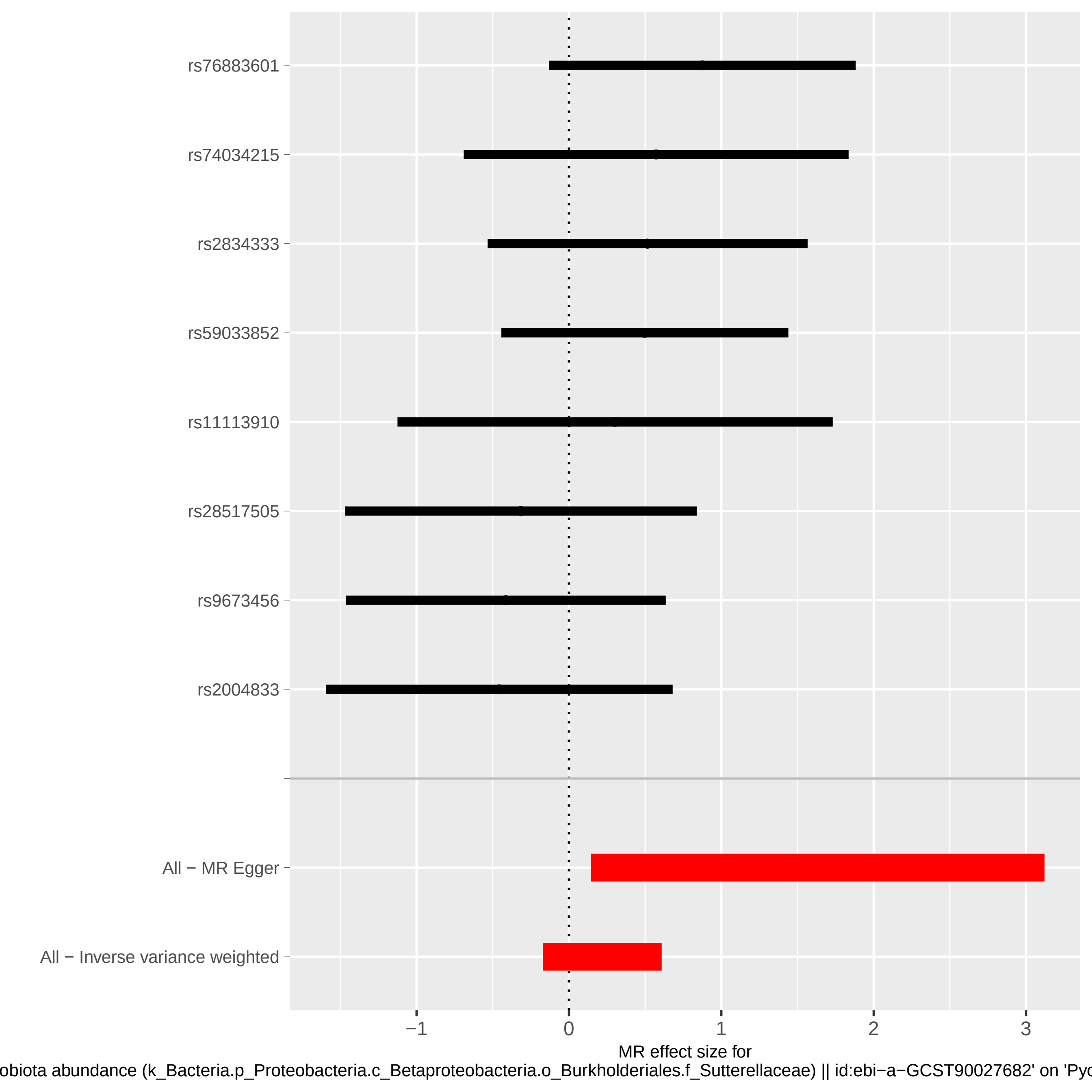

rs76883601
rs74034215
rs2834333
rs59033852
rs11113910
rs28517505
rs9673456
rs2004833
All − MR Egger
All − Inverse variance weighted
−1
0
1
2
3
MR effect size for
obiota abundance (k_Bacteria.p_Proteobacteria.c_Betaproteobacteria.o_Burkholderiales.f_Sutterellaceae) || id:ebi−a−GCST90027682' on 'Py
